# Editorial: Targeting cellular signalling pathways for disease therapy: the potential of cellular reprogramming and protein kinase inhibitors

**DOI:** 10.3389/fphar.2025.1580686

**Published:** 2025-03-19

**Authors:** Vanessa Marensi, Pedro Fontes Oliveira, Ariane Zamoner

**Affiliations:** ^1^ Department of Biochemistry, Cell and Systems Biology, Institute of Systems, Molecular and Integrative Biology, University of Liverpool, Liverpool, United Kingdom; ^2^ LAQV-REQUIMTE and Department of Chemistry, University of Aveiro, Aveiro, Portugal; ^3^ Laboratory of Biochemistry and Cell Signaling - LaBioSignal, Department of Biochemistry, Center of Biological Sciences, Federal University of Santa Catarina, Florianópolis, Santa Catarina, Brazil

**Keywords:** cancer, cell signal inhibitors, cellular reprograming, kinase, inflammation

Cellular reprogramming is particularly active during development, regeneration and cancer, when dynamic signaling pathways orchestrate cellular changes in response to input signals (Huyghe et al., 2024). It facilitates adaptation to exogenous pressure enabling tumor cells to adapt and survive in response to adverse microenvironments (Anderson and Simon, 2020; Hanahan and Weinberg, 2011). Understanding the mechanism these adaptations happen has fueled significant advances in medical science, particularly in regenerative medicine and targeted cancer therapies (He et al., 2021; Yan et al., 2024). Spatial-temporal integration of cellular signaling is fundamental to effective reprogramming. Dysregulation of protein kinases is key to the pathogenesis of various pathologies, particularly cancer, dictating disease progression and inhibitors targeting this family represent pivotal tools in modern therapeutics (Cohen, 2002). Transduction via MAPK and PI3K pathway has been extensively studied and is stablished as mediator of cancer signals (Corrales et al., 2022). Molecules targeting key signaling pathways like MAPK and PI3K providing tailored therapies have already been approved for clinical applications (Bahar et al., 2023; Dos Santos et al., 2022). Despite advances, cross-communication between these pathways occurs and further understanding is needed to uncover the mechanism signaling rewiring develops (Corrales et al., 2022). This Research Topic focuses on the rewiring signaling cascades as result of cellular reprograming and their target as therapeutic strategy consolidates recent advancements in understanding how cellular signaling pathways are modulated for therapeutic purposes. The studies included in this here provide a comprehensive overview of some aspects and developments in this field and are summarized in Figure 1.

**FIGURE 1:**
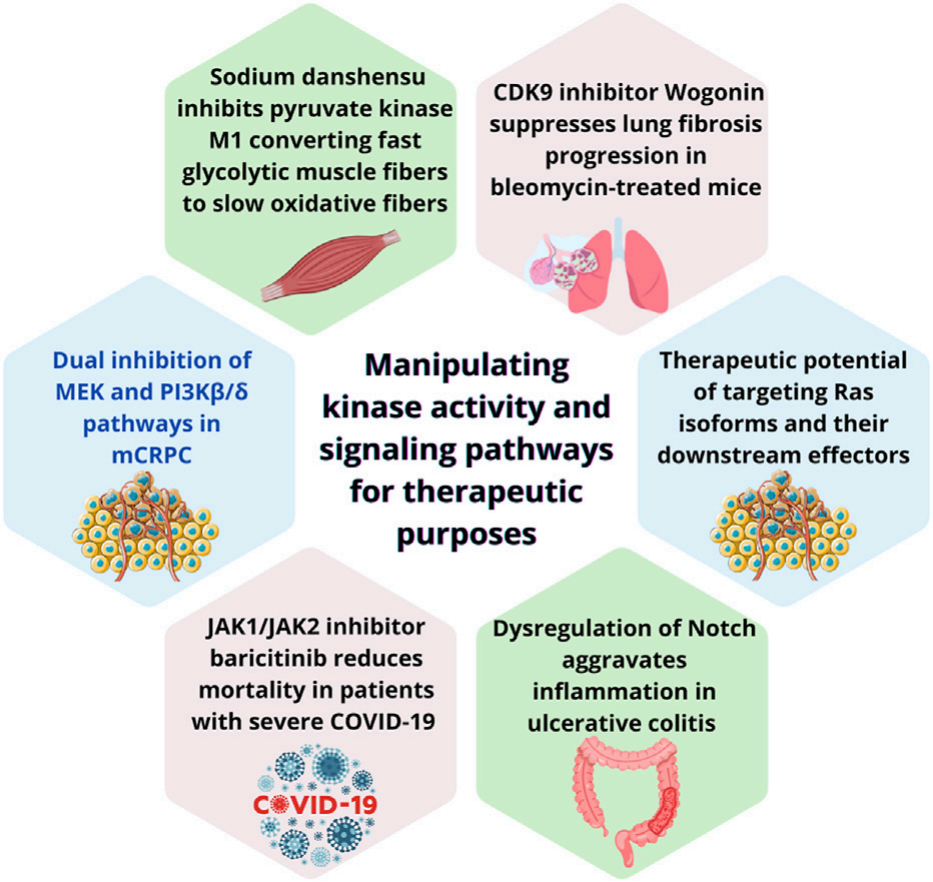
Summary of key findings from each study featured in this editorial, highlighting diverse therapeutic strategies targeting kinase activity and signaling pathways. (a) dual inhibition of MEK and PI3Kβ/δ pathways to overcome docetaxel resistance in metastatic castration-resistant prostate cancer (mCRPC); (b) Sodium danshensu‘s promotion of muscle fiber transformation by inhibiting pyruvate kinase M1, enhancing endurance and metabolic efficiency; (c) Wogonin‘s antifibrotic effects through CDK9 inhibition, reducing cellular senescence and fibrosis; (d) the therapeutic potential of targeting Ras isoforms and their downstream effectors, including advances in KRAS inhibitors; (e) the role of JAK1/JAK2 inhibitor baricitinib in reducing mortality among severe COVID-19 patients; and (f) dysregulation of the Notch signaling pathway in ulcerative colitis, revealing opportunities for pathway-specific interventions. Collectively, these studies highlight the transformative potential of manipulating kinase signaling in diverse disease contexts.

Ruiz de Porras et al. and Healy et al. explored the therapeutic potential of kinase inhibitor, offering valuable insights into their applications and mechanisms of action. Ruiz de Porras et al. show that MAPK and PI3K pathways are overactive in docetaxel-resistant cell lines. Dual inhibition of MEK1/2 (selumetinib) and PI3Kβ/δ (AZD8186) was sufficient to overcame resistance to docetaxel in metastatic castration-resistant prostate cancer (mCRPC). The combination reduced tumor growth and induced apoptosis in both *in vitro* and DU145-DR-derived xenograft mouse model expressing phosphatase and tensin homolog (PTEN) wild-type. This study shows the therapeutic potential of targeting MEK/ERK and PI3K/AKT crosstalk, which often drives resistance to monotherapies. The Ras family is a key regulator of cellular proliferation, survival, and differentiation known to mediate crosstalk between signaling cascades (Catozzi et al., 2022). Healy et al. provides a comprehensive review on Ras-mediated signaling activation in cancer and highlights the therapeutic potential of targeting Ras isoforms and their downstream effectors, particularly the MAPK and PI3K pathways. Ras mutations, particularly in KRAS, are prevalent in various cancers. A Gly to Cys mutation (KRAS-G12C) made mutated Ras a druggable target, revolutionizing the field. Nevertheless, challenges remain in addressing wild type Ras, other Ras mutations and associated drug resistance. By integrating biochemical, genomic, and proteomic information, current studies progress towards effective treatment and better outcomes for Rasopathies. Mao et al. work on a retrospective study investigate the use of kinase inhibitors in critical care settings. Baricitinib is a JAK1/JAK2 inhibitor, which use was shown to be associated with reduction of 28-day mortality in patients with severe COVID-19 requiring invasive mechanical ventilation. Intriguingly, patients in the baricitinib group were associated to increased hypertension and more likely to receive the antiviral drug nirmatrelvir and ritonavir.

In Zhang et al., the versatility of natural products as modulators of cellular signaling pathways was demonstrated by the effects of sodium danshensu (SDSS), a stable derivative of danshensu, on skeletal muscle fiber transformation. SDSS promoted the conversion of fast glycolytic muscle fibers to slow oxidative fibers by inhibiting pyruvate kinase M1 (PKM1), a kinase responsible for the generation of pyruvate and ATP during glycolysis. This shift enhances muscle endurance and metabolic efficiency in mice, providing new insights into the role of kinase modulation in muscle physiology. Both, SDSS-treated C2C12 myoblasts and mice exhibited increased oxidative capacity, improved glucose tolerance, and reduced markers of muscle atrophy. Wang et al. has also explored a compound of natural source. Wogonin, extracted from *Scutellaria baicalensis*, is a cyclin-dependent kinase 9 (CDK9) inhibitor and was shown to have an effective antifibrotic property in mice with bleomycin (BLM)-induced lung fibrosis, thereby mitigating its progression.

Notch signaling participates in various cellular processes, including the regulation of intestinal homeostasis. Ning et al. reviewed the role of notch pathway in ulcerative colitis, emphasizing how its dysregulation disrupts the balance of gut cell lineages, weakens the mucosal barrier, and aggravates inflammation. The complex architecture of this pathway creates a complex web of signaling transduction, including shifts in signaling as result of Notch cleavage by γ-secretase and the synergetic activity the doublecortin-like kinase 1 (DCLK1) in crypt epithelial cells or microorganism-specific signaling. Future therapeutic strategies could involve selective inhibition or activation of Notch components to achieve disease-specific outcomes, including the use of cleavage by γ-secretase inhibitors.

Despite the current progress, challenges remain in translating the current knowledge into clinical practice. The insights provided here pave the way for new studies, exploring alternative therapeutic strategies and the adaptability of cellular signaling pathways. They emphasize the potential of kinase inhibitors and pathway modulators in addressing a range of medical challenges, from cancer and fibrosis to metabolic diseases and inflammation.

## References

[B1] Anderson N. M. Simon M. C. (2020). The tumor microenvironment. Curr. Biol. 30 (16), R921-R925–r5. 10.1016/j.cub.2020.06.081 32810447 PMC8194051

[B2] Bahar M. E. Kim H. J. Kim D. R. (2023). Targeting the RAS/RAF/MAPK pathway for cancer therapy: from mechanism to clinical studies. Signal Transduct. Target. Ther. 8 (1), 455. 10.1038/s41392-023-01705-z 38105263 PMC10725898

[B3] Catozzi S. Ternet C. Gourrege A. Wynne K. Oliviero G. Kiel C. (2022). Reconstruction and analysis of a large-scale binary Ras-effector signaling network. Cell Commun. Signal. 20 (1), 24. 10.1186/s12964-022-00823-5 35246154 PMC8896392

[B4] Cohen P. (2002). Protein kinases—the major drug targets of the twenty-first century? Nat. Rev. Drug Discov. 1 (4), 309–315. 10.1038/nrd773 12120282

[B5] Corrales E. Levit-Zerdoun E. Metzger P. Mertes R. Lehmann A. Münch J. (2022). PI3K/AKT signaling allows for MAPK/ERK pathway independency mediating dedifferentiation-driven treatment resistance in melanoma. Cell Commun. Signal. 20 (1), 187. 10.1186/s12964-022-00989-y 36434616 PMC9700886

[B6] Dos Santos D. C. Rafique J. Saba S. Grinevicius V. Filho D. W. Zamoner A. (2022). IP-Se-06, a selenylated imidazo[1,2-*a*]pyridine, modulates intracellular redox state and causes akt/mTOR/HIF-1*α* and MAPK signaling inhibition, promoting Antiproliferative effect and apoptosis in Glioblastoma cells. Oxid. Med. Cell Longev. 2022, 3710449. 10.1155/2022/3710449 35360199 PMC8964227

[B7] Hanahan D. Weinberg R. A. (2011). Hallmarks of cancer: the next generation. Cell 144 (5), 646–674. 10.1016/j.cell.2011.02.013 21376230

[B8] He Y. Sun M. M. Zhang G. G. Yang J. Chen K. S. Xu W. W. (2021). Targeting PI3K/Akt signal transduction for cancer therapy. Signal Transduct. Target. Ther. 6 (1), 425. 10.1038/s41392-021-00828-5 34916492 PMC8677728

[B9] Huyghe A. Trajkova A. Lavial F. (2024). Cellular plasticity in reprogramming, rejuvenation and tumorigenesis: a pioneer TF perspective. Trends Cell Biol. 34 (3), 255–267. 10.1016/j.tcb.2023.07.013 37648593

[B10] Yan Z. Liu Y. Yuan Y. (2024). The plasticity of epithelial cells and its potential in the induced differentiation of gastric cancer. Cell Death Discov. 10 (1), 512. 10.1038/s41420-024-02275-x 39719478 PMC11668900

